# Enfermedad de Chagas, logros y perspectivas en Colombia

**Published:** 2022-06-01

**Authors:** Gabriel Parra-Henao, Mauricio Javier Vera

**Affiliations:** 1 Instituto Nacional de Salud, Bogotá, D.C. Instituto Nacional de Salud Bogotá, D.C.; 2 Ministerio de Salud y Protección Social, Bogotá, D.C. Ministerio de Salud y Protección Social Bogotá, D.C.

En la Región de las Américas, la enfermedad de Chagas es aún un reto de salud pública debido a que, por su naturaleza de zoonosis, no es posible erradicarla, y tiene diversos mecanismos de transmisión y ámbitos de presentación que exigen múltiples estrategias para su control y eliminación. En la región son tangibles los logros, especialmente en su interrupción, en la eliminación de la transmisión por las principales especies de vectores domiciliados y en el control de las transfusiones, alcanzados por los países bajo el liderazgo de la Organización Panamericana de la Salud (OPS). Dichos logros también se deben al gran aporte de la academia y la investigación en el marco subregional de las iniciativas de salud: la del Cono Sur (INCOSUR), la de los países andinos (IPA), la de los países de América Central (IPCA) y la de los países amazónicos para la vigilancia y control de la enfermedad de Chagas (AMCHA) ^1^.

La enfermedad de Chagas es endémica en 21 países latinoamericanos, aproximadamente, con 65 millones de personas en riesgo de contraería. Se estima que entre 6 y 7 millones de personas están infectadas en todo el mundo con el parásito *Trypanosoma cruzi* y la gran mayoría reside en Latinoamérica. Cada año se reportan más de 10.000 muertes relacionadas con esta enfermedad ^2^^-^^4^ que, cuando es sintomática, supone una carga financiera considerable para los sistemas de salud y las sociedades ^5^. Con un estimado de USD $ 690 millones en costos sanitarios y con pérdidas económicas anuales de USD $ 8 billones, la carga económica que impone la enfermedad de Chagas es igual o superior a la que provocan otras enfermedades infecciosas ^6^^,^^7^. Sin embargo, a pesar de su alta morbilidad y mortalidad, y de la importante carga económica asociada, tan solo el 7 % de las personas con la enfermedad ha sido diagnosticado y solo el 1 % recibe tratamiento etiológico ^8^. Su detección y el tratamiento oportuno tienen grandes beneficios, incluida la prevención de futuros casos de transmisión congénita en mujeres que no han recibido tratamiento, la curación serológica de bebés y niños, y la reducción de la evolución de la enfermedad hacia sus formas avanzadas en adultos ^9^^-^^13^, con la consecuente mejoría de su calidad de vida.

Podemos enmarcar el avance del conocimiento sobre la enfermedad de Chagas en el país en una serie de hitos: a partir del hallazgo de César Uribe Piedrahíta en 1929 de la presencia de *Trypanosoma cruzi* y *Trypanosoma rangeli* en ejemplares de la especie *Rhodnius prolixus* recolectados en el departamento del Tolima ^14^, se desarrollaron investigaciones puntuales en diferentes regiones del país con aportes al conocimiento de los vectores y la enfermedad; pero fue el trabajo de Augusto Corredor Arjona y sus colaboradores del Instituto Nacional de Salud, publicado en 1990 con el título “Distribución de los triatominos domiciliarios en Colombia” el que logró consolidar un conocimiento claro de la distribución en el país de los principales vectores de *T. cruzi*^15^. Por lo tanto, se considera el primer gran hito en la historia del estudio de esta enfermedad en el país, ya que permitió dimensionar su probable distribución en aquella época y permitió su inclusión en las actividades de la Unidad Administrativa Especial de Campañas Directas.

Después de estos trabajos pioneros, enfocados principalmente en los vectores, en 1995, el Ministerio de Salud expidió el Decreto 1738 mediante el cual se reglamentó la obligatoriedad del tamizaje de la infección por *T. cruzi* en todos los bancos de sangre del país ^16^, tamizaje que hoy tiene una cobertura del 100 % de las unidades de sangre, lográndose establecer una prevalencia por banco de sangre de alrededor del 0,3 %, lo que es un excelente logro y constituye el segundo hito histórico en el avance del control de la enfermedad de Chagas en Colombia.

En 1997, Colombia ingresó al Convenio Hipólito Unanue (CONHU), establecido por el Organismo Andino de Salud en la primera reunión de los países andinos sobre vectores de la enfermedad de Chagas realizada ese año en Bogotá. Allí se fijaron los compromisos de los países participantes frente al cumplimiento de los objetivos de control de la transmisión vectorial y transfusional de la tripanosomiasis americana en la región andina.

A partir del CONHU, en 1998, y con el auspicio del Ministerio de Salud, el país emprendió la tarea de formular el Programa Nacional de Prevención y Control de la Enfermedad de Chagas que, en su fase diagnóstica, cubrió una extensa área geográfica del país correspondiente a 15 departamentos, 539 municipios y 3.375 veredas. De esta manera, se logró determinar los factores de riesgo de infestación domiciliaria por triatominos en más de 41.000 viviendas y estudiar la seroprevalencia en más de 51.000 menores de edad.

En esa extensa encuesta financiada por el Ministerio de Salud y desarrollada por tres centros de investigación del país (ICMT, CINTROP y CIMPAT), con el apoyo y la participación del Instituto Nacional de Salud, se logró establecer que para esa época 124 municipios del país estaban en alto riesgo de transmisión y otros 194 tenían mediano riesgo. Dichos municipios se ubicaban principalmente en la región centro-oriental del país, en los departamentos de Arauca, Boyacá, Casanare, Cundinamarca, Meta, Norte de Santander, Santander y Tolima. Después, en un estudio de morbilidad de la enfermedad de Chagas en diferentes regiones, se establecieron en la Sierra Nevada de Santa Marta prevalencias de infección iguales o superiores a las halladas en los departamentos mencionados, lo que situó a esa region del país como una de alto riesgo de transmisión. Esta encuesta constituye el tercer hito histórico, pues dimensionó la magnitud del problema y generó el marco para la focalización de las intervenciones venideras.

A partir de los importantes aportes del programa nacional iniciado en 1998 y de la información recolectada, diferentes secretarías de salud departamentales se dieron a la tarea de continuar con algunas intervenciones de control de vectores que tuvieron algún impacto, pero que no constituían una estrategia integral de control vectorial.

Otro aspecto que debe tenerse en cuenta es que, conceptualmente, el programa nacional de la enfermedad de Chagas establecido en 1998 no se institucionalizó por su falta de relación con los planes sectoriales y sus metas nacionales, y de recursos, por lo que tampoco avanzó a las fases de ataque y consolidación. Se llega así al año 2008, cuando se formuló e implemento una estrategia de gestión integral, la cual permitió organizar, fortalecer y empoderar a los departamentos priorizados para abordar el problema, alinear la cooperación internacional y encaminar el país hacia la interrupción de la transmisión por vectores domiciliados en las zonas eco-epidemológicas, respetando la descentralización administrativa.

Paralelamente, en el 2011, se organizó la Red Chagas Colombia, iniciativa del entonces Colciencias, mediante la cual se unieron todos los grupos de investigación en la enfermedad de Chagas en el país, para generar conocimiento y tecnologías de calidad que oportunamente se transfirieron al subprograma postulado en el Ministerio de Salud para la enfermedad de Chagas, con lo que se contribuyó a su fortalecimiento.

A partir de los elementos aportados por la academia, la sociedad civil y la estrategia de gestión integral, en el 2013 se llegó al cuarto y principal hito histórico con el Plan Decenal de Salud Pública, en el cual se reconoció la enfermedad de Chagas como problema de salud pública priorizado, se establecieron metas para lograr la certificación de la interrupción y la reducción de la letalidad por casos agudos, se oficializó el subprograma nacional de prevención, control y eliminación de la enfermedad, y se asignaron recursos del presupuesto general de la nación, del Ministerio de Salud y de las direcciones territoriales de salud materializados en medicamentos, insecticidas, pruebas de diagnóstico, y talento humano, entre otros. De esta forma, la enfermad de Chagas adquirió relevancia en la planeación central y en las políticas territoriales, por medio de los planes de desarrollo y los acuerdos municipales y ordenanzas departamentales.

Si bien los avances en prevención y control han sido importantes, la atención médica de las personas infectadas por *T. cruzi* se ha quedado rezagada debido a las dificultades diagnósticas y terapéuticas que esta parasitosis sistémica ha entrañado.

Además de los hitos sucedidos en Colombia, debe mencionarse que la Organización Mundial de la Salud reconoció a la enfermedad de Chagas como una enfermedad tropical desatendida en el 2005. En su hoja de ruta para dichas enfermedades para el periodo de 2021 a 2030, se plantean cinco objetivos específicos:

1. Verificar la interrupción de la transmisión vectorial domiciliaria
2. Verificar la interrupción de la transmisión transfusional
3. Verificar de la interrupción de la transmisión por trasplante de órganos
4. Eliminar como problema de salud pública de la enfermedad de Chagas congénita
5. Suministrar del tratamiento antiparasitario al 75 % de la población elegible.

El país ha avanzado en los objetivos 1 y 2, pero aún tiene tareas pendientes en los objetivos 3 a 5. Sin embargo, es alentador el panorama de cumplimiento del objetivo 4, relativo a la transmisión congénita, por la inclusión de la enfermedad de Chagas en la estrategia de la "Eliminación de la Transmisión Materno Infantil" ETMI Plus y la aprobación por parte de la agencia financiadora UNITAID del proyecto de Comunidades Unidas para la Innovación, el Desarrollo y la Atención de la Enfermedad de Chagas, CUIDA Chagas (17), el cual se ejecutará en cuatro países de Suramérica (Bolivia, Brasil, Colombia y Paraguay). En nuestro país, este será ejecutado por el Ministerio de Salud y el Instituto Nacional de Salud mediante tres estudios independientes, pero interrelacionados:

1. uno sobre la implementación,
2. uno de validación de algoritmos de las pruebas de diagnóstico rápido, y
3. un estudio clínico doble ciego de fase III para evaluar un régimen más corto de tratamiento con benznidazol. El proyecto cuenta con el respaldo de los ministerios de salud de los cuatro países participantes y busca la colaboración activa de la OPS y la OMS para asegurar su alineamiento con la agenda internacional para la enfermedad de Chagas.

Los hitos descritos han jalonado el avance en el conocimiento y el control de la enfermedad de Chagas en Colombia. Ahora se tiene claridad sobre los diferentes factores que influyen en la presentación de la enfermedad en el país y la forma de abordarlos. Debemos resaltar la importancia de seguir consolidando el control de la enfermedad congénita, así como la necesidad de un mayor acceso al diagnóstico y el tratamiento; de estrategias de control en otras zonas del país como la Sierra Nevada de Santa Marta con una aproximación étnica y cultural a los pueblos indígenas; de la ampliación del conocimiento y la reglamentación para el estudio de los focos de la enfermedad de Chagas por transmisión oral, y de la implementación de estrategias de control de la transmisión por otras especies de vectores, lo que requiere la implementación de una estrategia de manejo integrado.

Consideramos que estas son las perspectivas que el país debe abordar para continuar con la ruta del control de la enfermedad de Chagas en los próximos años.
